# Painless Growing Swelling on the Palm of a 4-year-old Boy: Malignant Peripheral Nerve Sheath Tumor

**DOI:** 10.1055/s-0043-1771005

**Published:** 2023-10-24

**Authors:** Recep Öztürk, Kemal Kösemehmetoğlu, Fisun Ardıç Yükrük, Bedii Şafak Güngör

**Affiliations:** 1 Hospital de Treinamento e Pesquisa em Oncologia Dr. Abdurrahman Yurtaslan Ankara, Departamento de Ortopedia e Traumatologia, Ancara, Turquia; 2 Universidade Hacettepe, Faculdade de Medicina, Departamento de Patologia, Ancara, Turquia; 3 Hospital de Treinamento e Pesquisa em Oncologia Dr. Abdurrahman Yurtaslan Ankara, Departamento de Patologia, Ancara, Turquia

**Keywords:** child, diagnosis, differential, sarcoma, soft tissue neoplasms

## Abstract

In this study, we present a 4-year-old male patient with a slowly growing painless mass in the palm of his left hand for 2 years. Although musculoskeletal tumors are rare, hand localized tumors are even rarer in pediatric patients. The fact that very few (less than one in ten) tumors are malignant and there are dozens of subtypes, each with different treatment management, shows the importance of the management of these lesions. Appropriate diagnosis and management of soft tissue masses, especially insidious malignant tumors, is vital. Due to the rarity of soft tissue tumors, adequate guidelines for their management are limited. The purpose of this report is to present an example of the approach to one of the soft tissue tumors.

## Introduction

Soft tissue tumors are very rare and this creates a significant diagnostic dilemma. A biopsy may be useful in diagnosis, but it is usually insufficient for definitive sampling. The variability of cellularity on histopathological examination guides diagnoses from benign to malignant. The tumoral lesion may contain portions of cellular atypical neoplasm that may be missed by simple biopsy. 1 2 3

In this study, we present a 4-year-old male patient with a slowly growing painless mass in the palm of his left hand for 2 years. This report was presented to discuss the management of the case after taking the history and examining the radiological findings and specimen.

## Case Report

A 4-year-old boy was referred to our clinic with swelling on the left-hand volar face. In his history, his family stated that the child's hand had a lump the size of chickpea 2 years ago, and it was growing slowly. It was learned that in the examination 1 year ago, surgery was recommended in an external center, but the relatives of the patient did not accept it. He had no personal or family history of NF-1.

**Fig. 1:**
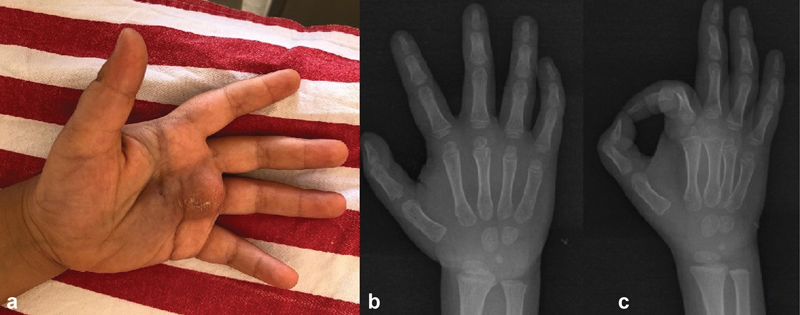
a) immobile, pasty soft tissue mass in the palm of the left hand. b,c) No pathological finding was detected in direct radiographs.

**Fig. 2:**
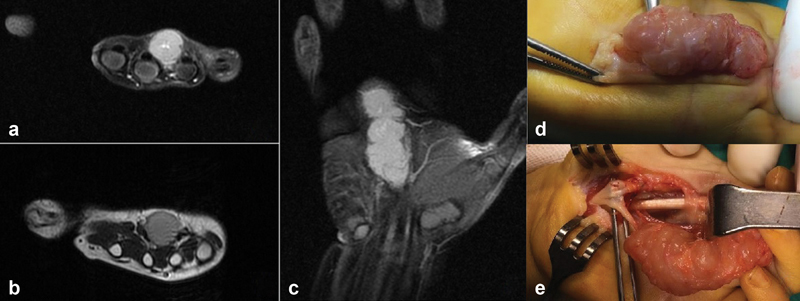
a,b,c) an MRI imaging, the lesion is approximately 4 × 1.5 × 2 cm in size, located between the 3rd and 4th metacarpals of the left-hand volar face, adjacent to the flexor tendon, hypointense in T1 sequence, hyperintense in T2 sequence. d,e) on intraoperative images, the lesion appears to involve the lateral branch of the median nerve.

In the clinical examination, there was an immobile, pulpy soft tissue mass in the palm of the left hand, slightly painful on palpation. The flexion of the involved fingers was limited due to the mass. This mass, which was painless, had mild pain with pressure on it. No pathological finding was found on direct radiographs ( Fig. 1 ). In MRI imaging, a lesion of approximately 4 × 1.5 × 2 cm in size, located between the 3rd and 4th metacarpals on the left-hand volar face, under the skin on the volar face, adjacent to the flexor tendon, hypointense in T1 sequence and hyperintense in T2 sequence was detected ( Fig. 2 ). True-cut biopsy was performed, and the diagnosis was considered as spindle cell lesion because of only caldesmon and focal actin positivity in the histopathological examination, no evidence of malignancy was found. What is your diagnosis?

Total excision was planned for the patient. In the intraoperative evaluation, it was seen that the tumor was limited to the capsule, the median nerve was divided into branches at the tumor level, and its lateral branch was intertwined with the tumor ( Fig. 2 ). The lateral branch of the median nerve was also sacrificed and the tumor was totally excised.

**Fig. 3:**
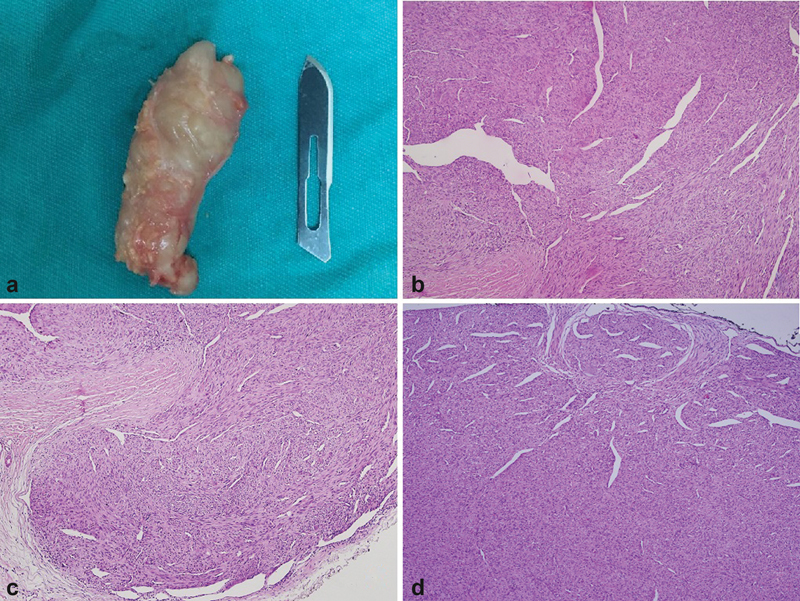
a) On macroscopic examination, a fusiform soft tissue mass of 5 × 2 × 2 cm, relatively well-circumscribed, creamy white in color, and hard consistency. b,c: Spindle-shaped cells arranged in bundles and fascicles with wavy nuclei in an eosinophilic cytoplasm. Peripheral nerve bundles are observed peripherally. d: Low power view of a low-grade hemangiopericytoma-like MPNST with typical branching vascular pattern.

Macroscopic examination revealed a fusiform soft tissue mass measuring 5 × 2 × 2 cm was relatively well-circumscribed, cream-white in color, and hard consistency. It seemed that it has a relationship with peripheral nerves ( Fig. 3a ).

**Fig. 4:**
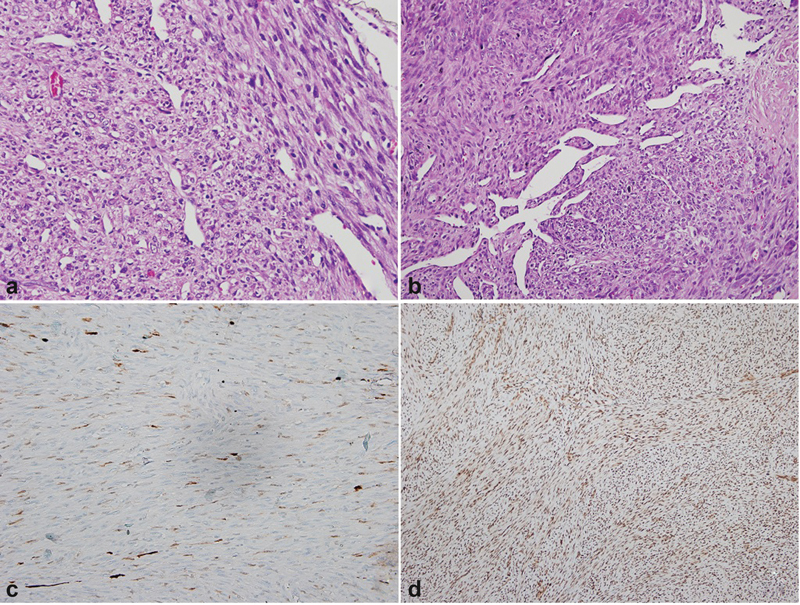
a,b) Mitotic rate was observed as 3-4/10 HPF. c): Patcynuclea rand cytoplasmic S100 immunexpression (S100 × 400). d) Nuclear INI 1 immunoreactivity.

Microscopically, the mass showed spindle-shaped cells arranged in bundles and fascicles with wavy nuclei in an eosinophilic cytoplasm. Peripheral nerve bundles were observed peripherally ( Fig. 3b,c ). Low-grade hemangiopericytoma-like areas were seen throughout the tumor with a typical branching vascular pattern ( Fig. 3d ). Mitotic rate was observed as 3-4/10 HPF ( Fig. 4a,b ) and 50 or more HPFs examined before a definitive decision was made. No tumor necrosis and hemorrhage were observed.

An extensive immunohistochemistry study was made including S100, SOX 10, NSE, CD163, B-catenin, CD57, CD99, CD34, actin, desmin, Myo D1, myogenin, calponin, lysozyme, ERG, Fli 1, TLE-1, EMA, INI-1, bcl2, CD57 in order to exclude other spindle cell sarcoma.

The tumor was immunoreactive for S-100 focally/ patchy. Ki 67 proliferative activity was in the range of 5-8%. There was no INI-1 loss ( Fig. 4c,d ). CD 99 revealed cytoplasmic dot-like staining.

The diagnosis of low-grade malignant peripheral nerve sheath tumor (MPNST) with a hemangiopericytoma-like vascular pattern was made because of the macroscopically fusiform enlargement of the mass, its relationship with the peripheral nerve, mild atypia of neoplastic cells, mitotic activity, immunohistochemically S100 focal positivity, and hemangiopericytoma-like vascular pattern.Resection of the tumor bed was performed.

The histopathological diagnosis was based on Schwann cell differentiation and exclusion of other spindle cell sarcoma.

**Fig. 5:**
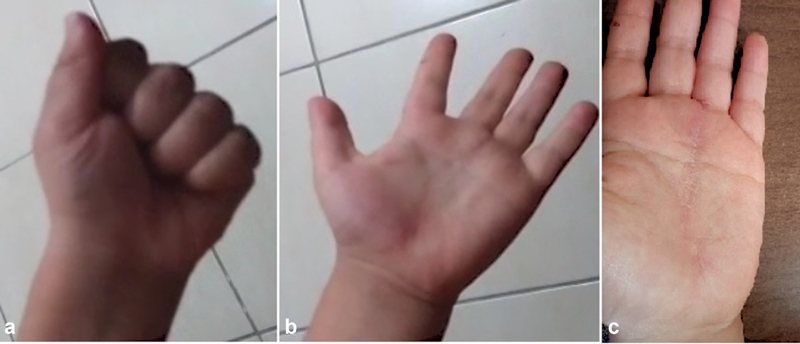
The patient is under recurrence-free follow-up at 18 months postoperatively, hand functions are excellent.

Post-operative neurovascular examination of the patient was normal. The case was discussed by the multidisciplinary team. Considering that the tumor was low grade and completely removed, follow-up without adjuvant treatment was planned. The patient is at 18 months with excellent hand functions ( Fig. 5 ), recurrence-free follow-up.

## Discussion

Hand-located peripheral nerve sheath tumors are rare and often benign. Malignant peripheral nerve sheath tumors (MPNST), or malignant schwannoma, originate from neuroepithelial tissue and constitute approximately 5% of soft tissue sarcomas. 4 Hand-held MPNSTs are usually in the form of case reports or a small number of case series. 2 5

Up to 50% of MPNSTs occur in neurofibromatosis-1 (NF-1) patients. Preoperative clinical diagnosis of MPNST is difficult, especially outside the context of NF-1. Clinical features include an enlarging soft tissue mass, usually in continuity with the main nerve trunk of the upper or lower extremity. 3 6 In our case, the patient did not have a history of NF, and there was no clinical finding other than a mass in the hand of the young child.

Hand-located MPNSTs are particularly difficult to diagnose and are often misdiagnosed. Wood et al. 7 reported a case of malignant schwannoma originating from the median nerve, which was misdiagnosed as carpal tunnel syndrome. In the case reported by Devnani et al., 2 the tumor was initially misdiagnosed as Ewing's sarcoma and the patient was given induction chemotherapy. In our case, there was no evidence of malignancy in the histopathological examination of the biopsy material taken at the beginning, but sarcoma was diagnosed after excision. It required surgery for the resection of the tumor bed.

This case highlights the need for a surgeon to be vigilant, the need to take a biopsy, and the need for extensive resection of a potentially malignant mass. In addition, treatment management, especially in sarcomas, must be provided by a multidisciplinary team.
