# Supplementary material for: Painless Growing Swelling on the Palm of a 4-year-old Boy: Malignant Peripheral Nerve Sheath Tumor
Source: Rev Bras Ortop (Sao Paulo). 2023 Oct 24;59(Suppl 1):e104–8. doi: 10.1055/s-0043-1771005 (PMC11254434; doi:10.1055/s-0043-1771005)
Supplement: Related texts

# Research article: Inchaço crescente indolor na palma da mão de um menino de 4 anos: Tumor maligno da bainha do nervo periférico

**Keywords:** Criança, Diagnóstico diferencial, Neoplasias de tecidos moles, Sarcoma

## Abstract

Neste estudo, apresentamos um paciente do sexo masculino de 4 anos com uma massa indolor de crescimento lento na palma da mão esquerda há 2 anos. Embora os tumores musculoesqueléticos sejam raros, os tumores localizados nas mãos são ainda mais raros em pacientes pediátricos. O fato de pouquíssimos (menos de um em cada dez) tumores serem malignos e existirem dezenas de subtipos, cada um com tratamento diferente, mostra a importância do manejo dessas lesões. O diagnóstico e manejo apropriados de massas de tecidos moles, especialmente tumores malignos insidiosos, são vitais. Devido à raridade dos tumores de tecidos moles, as diretrizes adequadas para seu manejo são limitadas. O objetivo deste relato é apresentar um exemplo de abordagem de um dos tumores de partes moles.

## Introdução

Os tumores de tecidos moles são muito raros e isso cria um dilema diagnóstico significativo. Uma biópsia pode ser útil no diagnóstico, mas geralmente é insuficiente para uma amostragem definitiva. A variabilidade da celularidade no exame histopatológico orienta diagnósticos de benignos a malignos. A lesão tumoral pode conter porções de neoplasia celular atípica que podem passar despercebidas por biópsia simples. 1 2 3

Neste estudo, apresentamos um paciente do sexo masculino de 4 anos com uma massa indolor de crescimento lento na palma da mão esquerda há 2 anos. Este estudo foi apresentado para discutir o manejo do caso após colher o histórico e examinar os achados radiológicos e os de espécime.

## Relato de caso

Um menino de 4 anos foi encaminhado à nossa clínica com edema na face volar esquerda. Em seu histórico, sua família afirmou que a mão da criança tinha um caroço do tamanho de grão de bico há 2 anos, e estava crescendo lentamente. Soube-se que no exame de 1 ano atrás foi indicada cirurgia em centro externo, mas os familiares da paciente não aceitaram. Ele não tinha histórico pessoal ou familiar de NF1.

Ao exame clínico, havia massa de partes moles, imóvel, pulposa, na palma da mão esquerda, levemente dolorosa à palpação. A flexão dos dedos envolvidos era limitada pela massa. Essa massa, que era indolor, apresentava dor leve com pressão. Nenhum achado patológico foi encontrado em radiografias diretas ( Fig. 1 ). Na RM, lesão de aproximadamente 4 × 1,5 × 2 cm, localizada entre o 3° e o 4° metacarpos da face volar esquerda, sob a pele da face volar, adjacente ao tendão flexor, hipointensa em sequência T1 e hiperintensa na sequência T2 foi detectada ( Fig. 2 ). Foi realizada true-cut biópsia, e o diagnóstico foi considerado lesão fusiforme, uma vez que apenas caldesmon e actina focal testaram positiva no exame histopatológico, e não foi encontrada evidência de malignidade. Qual seria o seu diagnóstico?

**Fig. 1:** a) massa de partes moles, pastosa e imóvel na palma da mão esquerda. b,c) Nenhum achado patológico foi detectado nas radiografias diretas.

**Fig. 2:** a,b,c) na RM, a lesão mede aproximadamente 4 × 1,5 × 2 cm, localizada entre o 3° e o 4° metacarpos da face volar esquerda, adjacente ao tendão flexor, hipointensa na sequência T1, hiperintensa na sequência T2. d,e) nas imagens intraoperatórias, a lesão parece envolver o ramo lateral do nervo mediano.

A excisão total foi planejada. Na avaliação intraoperatória, observou-se que o tumor limitava-se à cápsula, o nervo mediano se dividia em ramos ao nível do tumor e seu ramo lateral se entrelaçava com o tumor ( Fig. 2 ). O ramo lateral do nervo mediano também foi sacrificado e o tumor foi totalmente excisado.

O exame macroscópico revelou uma massa fusiforme de tecidos moles medindo 5 × 2 × 2 cm, relativamente bem circunscrita, de cor branco-creme e consistência dura. Parece que tem relação com nervos periféricos ( Fig. 3a ).

**Fig. 3:** a) ao exame macroscópico, massa fusiforme de partes moles de 5 × 2 × 2 cm, relativamente bem circunscrita, de coloração branco-creme e consistência dura. b,c: Células fusiformes dispostas em feixes e fascículos com núcleos ondulados em citoplasma eosinofílico. Os feixes de nervos periféricos são observados perifericamente. d: Visão de baixo aumento de um TMBNP semelhante a hemangiopericitoma de baixo grau com padrão vascular ramificado típico.

Microscopicamente, a massa apresentava células fusiformes dispostas em feixes e fascículos com núcleos ondulados em citoplasma eosinofílico. Feixes de nervos periféricos foram observados perifericamente ( Fig. 3b,c ). Áreas semelhantes a hemangiopericitoma de baixo grau foram observadas em todo o tumor com um padrão vascular ramificado típico ( Fig. 3d ). A taxa mitótica foi observada como 3-4/10 HPF ( Fig. 4a,b ) e 50 ou mais HPFs foram examinados antes que uma decisão definitiva fosse tomada. Não foram observadas necrose tumoral e hemorragia.

**Fig. 4:** a,b) A taxa mitótica foi observada como 3-4/10 HPF. c): Imunoexpressão patcinuclear e citoplasmática S100 (S100 × 400). d) Imunorreatividade nuclear INI 1.

Um extenso estudo imunohistoquímico foi feito incluindo S100, SOX 10, NSE, CD163, B-catenina, CD57, CD99, CD34, actina, desmina, Myo D1, miogenina, calponina, lisozima, ERG, Fli 1, TLE-1, EMA, INI-1, bcl2, CD57 para excluir outros sarcomas de células fusiformes.

O tumor era imunorreativo para S-100 focalmente/desigual. A atividade proliferativa do Ki 67 estava na faixa de 5-8%. Não houve perda de INI-1 ( Fig. 4c,d ). O CD99 revelou coloração semelhante a um ponto citoplasmático.

O diagnóstico de tumor maligno da bainha do nervo periférico (TMBNP) de baixo grau com padrão vascular semelhante a hemangiopericitoma foi feito devido ao alargamento macroscopicamente fusiforme da massa, sua relação com o nervo periférico, leve atipia de células neoplásicas, atividade mitótica, imunohistoquimicamente positividade focal S100 e padrão vascular semelhante a hemangiopericitoma. Foi realizada ressecção do leito tumoral.

O diagnóstico histopatológico foi baseado na diferenciação das células de Schwann e exclusão de outros sarcomas de células fusiformes.

O exame neurovascular pós-operatório do paciente foi normal. O caso foi discutido pela equipe multidisciplinar. Considerando que o tumor era de baixo grau e completamente removido, foi planejado seguimento sem tratamento adjuvante. O paciente está com 18 meses com excelentes funções manuais ( Fig. 5 ), acompanhamento sem recorrência.

**Fig. 5:** O paciente encontra-se em acompanhamento sem recidiva aos 18 meses de pós-operatório, as funções da mão são excelentes.

## Discussão

Os tumores da bainha dos nervos periféricos localizados na mão são raros e geralmente benignos. Os tumores malignos da bainha do nervo periférico (TMBNP), ou schwannoma maligno, originam-se do tecido neuroepitelial e constituem aproximadamente 5% dos sarcomas de partes moles. 4 TMBNPs na mão são geralmente relatados na forma de relatos de casos ou um pequeno número de séries de casos. 2 5

Até 50% dos TMBNPs ocorrem em pacientes com neurofibromatose-1 (NF-1). O diagnóstico clínico pré-operatório de TMBNP é difícil, especialmente fora do contexto da NF-1. As características clínicas incluem uma massa crescente de tecidos moles, geralmente em continuidade com o tronco nervoso principal da extremidade superior ou inferior. 3 6 Em nosso caso, o paciente não tinha história de NF e não havia outro achado clínico além de uma massa na mão da criança.

TMBNPs localizados na mão são particularmente difíceis de diagnosticar e muitas vezes são diagnosticados erroneamente. Wood et al. 7 relataram um caso de schwannoma maligno originário do nervo mediano, que foi diagnosticado erroneamente como síndrome do túnel do carpo. No caso relatado por Devnani et al., 2 o tumor foi inicialmente diagnosticado erroneamente como sarcoma de Ewing e o paciente recebeu quimioterapia de indução. Em nosso caso, não houve evidência de malignidade no exame histopatológico do material de biópsia coletado no início, mas o sarcoma foi diagnosticado após a excisão. Houve necessidade de cirurgia para ressecção do leito tumoral.

Este caso destaca a necessidade de um cirurgião estar vigilante, a necessidade de fazer uma biópsia e a necessidade de ressecção extensa de uma massa potencialmente maligna. Além disso, o manejo do tratamento, principalmente nos sarcomas, deve ser feito por uma equipe multidisciplinar.
